# Benchmarking Selected Density Functionals and Dispersion Corrections for MOF‑5 and Its Derivatives

**DOI:** 10.1021/acs.jctc.5c00399

**Published:** 2025-07-14

**Authors:** Joshua Edzards, Julia Santana-Andreo, Holger-Dietrich Saßnick, Caterina Cocchi

**Affiliations:** † 11233 Carl von Ossietzky Universität Oldenburg, Institute of Physics, 26129 Oldenburg, Germany; ‡ Friedrich-Schiller Universität Jena, Institute for Condensed Matter Theory and Optics, 07743 Jena, Germany

## Abstract

Accurate computational predictions of metal–organic frameworks (MOFs) and their properties are crucial for discovering optimal compositions and applying them in relevant technological areas. This work benchmarks density functional theory approaches, including semilocal, meta-GGA, and hybrid functionals with various dispersion corrections, on MOF-5 and three of its computationally predicted derivatives, analyzing structural, electronic, and vibrational properties. Our results underline the importance of explicitly treating van der Waals interactions for an accurate description of structural and vibrational properties and indicate the meta-GGA functional R2SCAN as the best balance between accuracy and efficiency for characterizing the electronic structure of these systems, in view of future high-throughput screening studies on MOFs.

## Graphical abstract

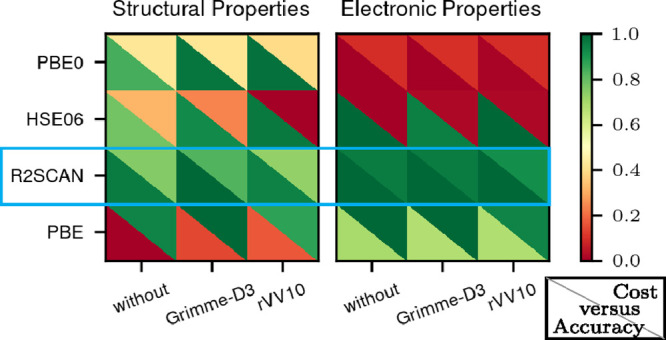

## 1 Introduction

Metal–organic frameworks (MOFs) are an established class of materials formed by an organic network connected by metal nodes.^1–4^ Their structural flexibility and chemical versatility have brought them under the spotlight in crucial technological areas, including energy conversion and storage, as well as gas sensing and adsorption.^5–10^ The current experimental efforts aimed at developing sustainable recipes for the synthesis of MOFs^11,12^ are mirrored by the implementation of efficient computational schemes that are suitable not only for simulating the fundamental properties of existing systems^13–16^ but also for predicting new compositions and crystal structures.^17–20^

High-throughput screening calculations based on first-principles methods have shown their potential in exploring the configurational space of technologically relevant material classes, ranging from semiconducting photocathodes^21–26^ to halide perovskites,^27–30^ and from thermoelectrics^31–33^ to complex oxides for batteries.^34,35^ These studies have contributed to expanding the range of available compounds for specific applications and paved the way for discovering new materials. The predictive power of ab initio high-throughput screening approaches is, however, crucially influenced by the accuracy of the underlying approximations. In particular, the performance of density functional theory (DFT), the workhorse method for quantum-mechanical simulations in computational materials science, is extremely susceptible to the adopted exchange-correlation functional. While the extensive use of DFT has led to practical recipes and common knowledge for the most established materials,^36,37^ extensive benchmarks are mandatory for hybrid systems^38,39^ and new compounds.^40–42^

In this work, we investigate the performance of different flavors of exchange-correlation functionals and dispersion correction schemes on the structural, electronic, and vibrational properties of MOF-5, a prototypical metal–organic framework,^43^ and three of its variants with a Sr metal node in place of Zn and/or hydroxyl-functionalized organic linkers, identified as energetically stable in a recent high-throughput screening study based on DFT.^19^ We examine how the generalized gradient approximation (GGA) in the Perdew–Burke–Ernzerhof (PBE) implementation,^44^ the metaGGA functional R2SCAN,^45^ and the hybrid functionals PBE0 ^46^ and HSE06 ^47,48^ predict lattice parameters, bond lengths, Bader charges, electronic gaps, density of states, and phonon dispersions in MOF-5. Two schemes for dealing with van der Waals (vdW) interactions are considered, and their performance is contrasted against plain functionals, not including any treatment of dispersion corrections. The obtained results and a thorough analysis of the computational costs reveal that R2SCAN provides the optimal trade-off between accuracy and numerical efforts, and that accounting for vdW interactions is crucial to properly describe the structural and vibrational properties of these systems.

## 2 Methods and Systems

### 2.1 Theoretical Background

This work is based on DFT^49^ in the Kohn–Sham (KS) framework,^50^ where the effective single-particle Schrödinger equation is solved for each (valence) electron in the system. The KS Hamiltonian comprises the kinetic energy operator and the effective potential per particle *v*_eff_ = *v*_ext_ + *v*_H_ + *v*_xc_, where the external potential *v*_ext_ describes the interaction between electrons and nuclei, the Hartree potential *v*_H_ the repulsion experienced by each electron from the surrounding electron density, and the exchange-correction (xc) potential *v*_xc_ all the remaining electron–electron interactions. Since the exact form of *v*_xc_ is unknown, this term has to be approximated, and the adopted recipe ultimately determines the accuracy of the results.

Approximations for *v*_xc_ are ranked according to the dependence of the xc energy on the density. The so-called local-density approximation introduced by Kohn and Sham^50^ represents the lowest rung. At the next level, we find the GGA, where *v*_xc_ is determined from the local electron density and its gradient. In this study, we utilize the PBE^44^ parametrization of GGA. Further refinements of *v*_xc_ incorporate the kinetic energy density, i.e., the second derivative of the density, leading to the so-called meta-GGA functionals. Here, we adopt R2SCAN,^45^ a well-established representative of metaGGA functionals. Hybrid functionals include a fraction of Hartree–Fock exchange to enhance the accuracy of the band gaps and mitigate the self-interaction error, which plagues all pure DFT functionals. Notable examples of hybrid functionals are the *global hybrid* PBE0,^46^ incorporating 25% of exact exchange, and the *range-separated* hybrid functional HSE06,^47,48^ including a short-range Hartree–Fock exchange component and a long-range component that aligns with the PBE functional. Both functionals are employed in this work. Since the calculation of the Hartree–Fock exchange is particularly demanding, due to the nonlocal nature of the integrals involved, the application of an auxiliary density matrix method can reduce the costs without compromising on accuracy.^51,52^

Long-range and weak interactions, such as vdW forces, are not accurately captured by standard DFT functionals. To address this limitation, an additional term is introduced into the effective potential. A widely adopted and numerically inexpensive method is the so-called Grimme-D3 ^53^ correction, which empirically models dispersion forces and enhances the accuracy of DFT calculations for systems where such interactions are significant. A more sophisticated recipe is provided by rVV10,^54^ where a nonlocal vdW correction is embedded into the xc functional. Both approaches are considered in this work.

### 2.2 Computational Details

All calculations performed in this work are carried out with CP2K^55^ (version 2024.1) employing Goedecker-Teter-Hutter pseudopotentials.^56^ A 2 × 2 × 2 k-mesh generated with the Monkhorst–Pack scheme^57^ is used in all runs in conjunction with cutoff and relative cutoff energies of 600 and 100 Ry, respectively. The PBE, R2SCAN, PBE0, and HSE06 approximations for *v*_xc_ are adopted in this work in conjunction with the Grimme-D3 and the rVV10 schemes for dispersion corrections, both adjusted to the corresponding functional. For Grimme-D3, the default settings for PBE are used also for PBE0, and HSE06, while for R2SCAN, the corresponding parameters are taken. For rVV10, the parameter *C* = 0.0093 remains fixed while *b* is set to 10.0 ^58,59^ for PBE, PBE0, and HSE06, and to 11.95 ^60^ for R2SCAN. The MOLOPT triple-ζ basis set with double polarization is applied for all simulations, except for the preoptimization phase, where a double-ζ basis set with single polarization is utilized along with pressure and force thresholds of 200 bar and 0.005 Ha/bohr, respectively. For the volume optimization, these parameters are tightened to 100 bar and 0.0005 Ha/bohr, respectively, while maintaining fixed angles to uphold the symmetry of the initial structure. To compute phonon frequencies, the cutoff and relative cutoff energies are increased to 2200 and 500 Ry for PBE and PBE0, and to 3200 and 600 Ry for R2SCAN calculations. Additionally, for this task, the pressure and force thresholds are reduced to 100 bar and 1.9 × 10^–5^ Ha/bohr, respectively.

The density of states (DOS) is computed in a 2 × 2 × 2 supercell to mimic a corresponding k-point sampling. The Bader theory,^61,62^ which has been successfully applied on MOFs,^16,19,20^ is applied using the package Critic2 ^63^ (version 1.1) alongside the Yu-Trinkle integration technique.^64^ The harmonic interatomic force constants are calculated using Phonopy,^65,66^ which applies finite displacements, here chosen with an amplitude of 0.01 Å, to systematically perturb the atomic positions. To compute the vibrational response of the MOFs, 19 2 × 2 × 2 supercells, each containing 848 atoms, are generated from the primitive cell preserving its *Fm*3̅*m* cubic space group. For the phonon calculations with the PBE0 functional, which are considerably more demanding than those with PBE and R2SCAN, unit cells (1 × 1 × 1) are used. In the case of Sr-MOF-5, a conventional cell containing 424 atoms is needed to accurately compute the force constants in this setup. The structures and plots are produced with the in-house implemented Python package aim2dat.^67^

Calculations are performed on infrastructure provided by the German National High Performance Computing Alliance (Emmy supercomputer in Göttingen) on Intel Sapphire Rapids Xeon Platinum 8468 double nodes, with a base clock frequency of 2.10 GHz, a maximum turbo clock frequency of 3.80 GHz, and 48 cores each. In the computational resources analysis, the number of core hours is calculated as 96 × *n* × *t*, where 96 is the total number of cores per node, *n* the total number of nodes, and *t* the total runtime in hours.

### 2.3 Construction of the Systems

The systems considered in this work are MOF-5 and derivatives in the structure proposed by Butler et al.,^68,69^ characterized by a face-centered cubic lattice (*Fm*3̅*m* space group) with an experimental lattice constant of *a* = 25.87 Å.^70^ MOF-5 is characterized by Zn atoms interconnected via 1,4-benzene-dicarboxylate (BDC) linkers (Figure 1a). In earlier work, we computationally explored MOF-5 variants with different metal nodes and/or functionalized linker, identifying a set of energetically stable structures.^19^ Here, we consider four of such systems with Zn and Sr modes and with the BDC either H-passivated or functionalized with two OH groups in diagonal positions (Figure 1b). The primitive cells with (without) OH-functionalization include 118 (106) atoms in total.

**Figure 1:**
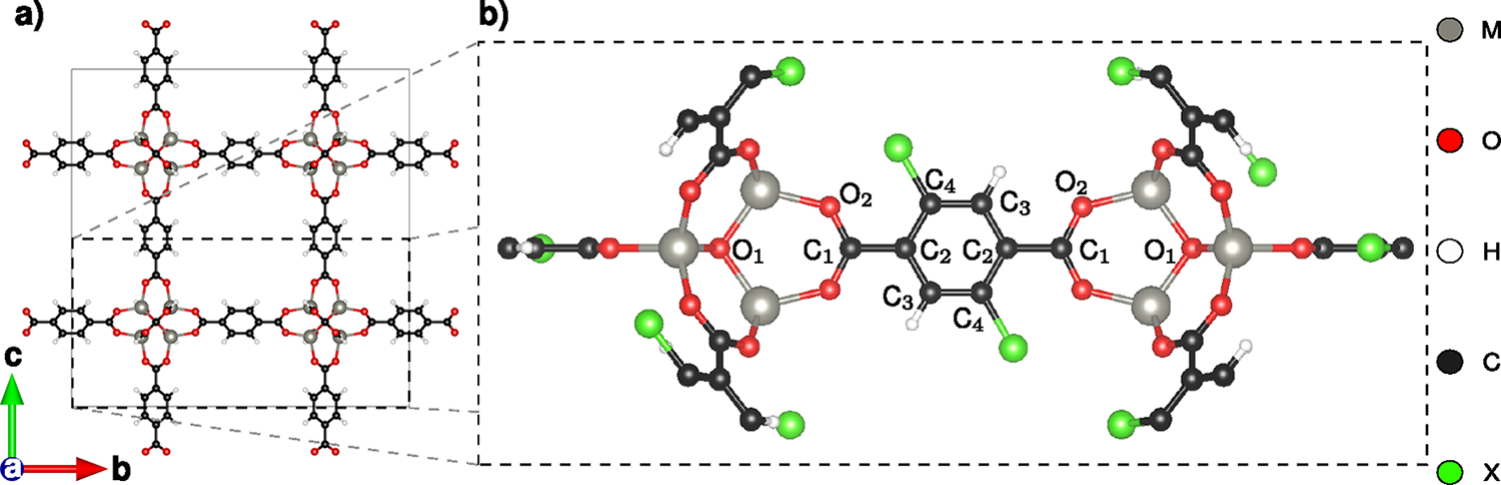
(a) Conventional cubic cell of MOF-5 and (b) zoom-in on the functionalized linker molecule in the coordinated framework with diagonal functionalization (X = H, OH). Zn and Sr are considered as metal nodes (M). The labels assigned to carbon and oxygen atoms are used in the analysis.

## 3 Results

### 3.1 Structural Properties

We begin our analysis by discussing the influence of exchange-correlation (xc) functionals and dispersion corrections on the structural properties of the MOFs, focusing initially on the lattice parameter *a*. For conventional MOF-5, the adopted *v*_xc_ approximation strongly affects the crystal volume (Figure 2a). Due to its known underbinding behavior,^71^ the PBE functional overestimates the lattice constant *a*. While the results are improved by incorporating dispersion corrections, the PBE lattice constant exceeds the experimental value^70^ by more than 0.2 Å independent of the treatment of vdW forces (Table S1). In contrast, hybrid functionals and R2SCAN, when combined with the Grimme-D3 correction, yield lattice parameters in agreement with experiment^70^ (Figure 2a, left) differing by less than 0.02 Å. Neglecting dispersion corrections slightly overestimates *a* for both hybrid functionals and R2SCAN. In the latter, the difference between the Grimme-D3-corrected result and the uncorrected one is only 0.02 Å. On the other hand, rVV10 leads to an underestimation of the lattice parameter which is most pronounced with R2SCAN and least so with HSE06. Similar trends are obtained with PBE0 (Figure 2a, left). The excellent prediction of the experimental lattice parameter provided by R2SCAN is evident from the root-mean-square error (RMSE) of <0.015 Å obtained for MOF-5, for which an experimental reference is available,^70^ see Table S4. RMSE values below or close to 0.057 Å are yielded by PBE0 and HSE06, respectively, while PBE features errors between 0.211 and 0.253 Å depending on the adopted dispersion correction. The addition of hydroxyl groups to the BDC linker does not qualitatively change the crystal structure (Figure 2a, right). PBE notably overestimates the lattice constant compared to all other functionals tested. The hybrid functionals, HSE06 and PBE0, predict larger volumes than R2SCAN. Including dispersion corrections reduces *a*, with a more pronounced effect with rVV10 than with the Grimme-D3 method.

**Figure 2:**
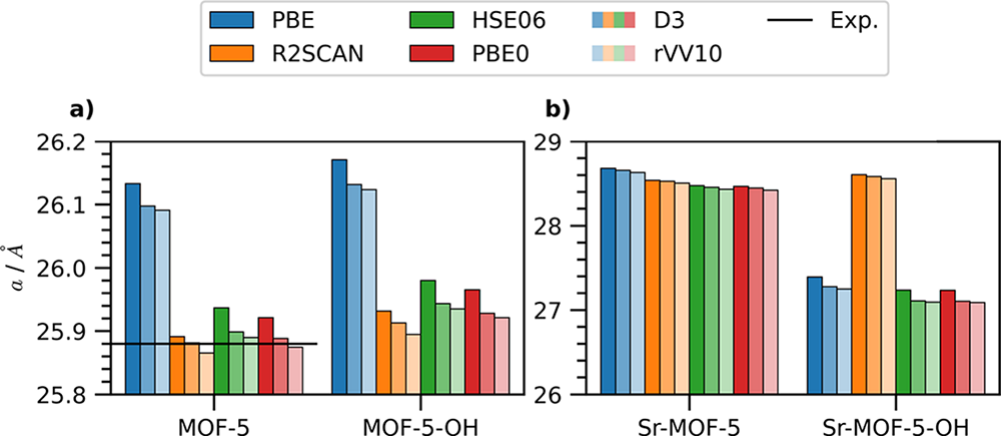
Lattice parameter *a* of (a) conventional MOF-5 and (b) its Sr-substituted counterpart with H-passivated (left) and hydroxyl functionalized BDC (right). The experimental reference available for conventional MOF-5 ^70^ is indicated by a horizontal bar.

Similar trends are obtained for the Sr-based MOF-5 with H-passivation (Figure 2b, left). PBE yields a larger lattice parameter compared to the hybrid and meta-GGA functionals. Incorporating vdW interactions using the Grimme-D3 and the rVV10 methods systematically reduces *a* across all tested functionals. The variations obtained with different *v*_xc_ and vdW treatments are consistent with the results for pristine MOF-5, see Table S1. Note the significantly larger scale of the *y*-axis in Figure 2b compared to Figure 2a.

The same analysis performed on the OH-functionalized Sr-based MOF-5 reveals qualitatively different results, see Figure 2b, right. R2SCAN predicts the largest lattice constant, which is reduced by approximately 1.1 Å by adopting PBE (Table S1). The two hybrid functionals, HSE06 and PBE0, yield nearly identical lattice constants, smaller than the PBE value. The trend for vdW corrections is consistent with the other compounds: including either the Grimme-D3 and rVV10 treatment systematically reduces *a* compared to the uncorrected case. Since experimental data for this predicted structure are missing, we can only compare our computational results. As discussed in ref 19, hydroxyl functionalization induces a significant torsion (approximately 15°) in the BDC linker of the Sr-based MOF-5. In the resulting structure, the oxoclusters are significantly distorted, leading to a corresponding decrease in the unit cell volume compared to the H-passivated counterpart.^19^ While all functionals capture this effect (Figure 2b), R2SCAN converges to a different structural minimum with a different cell size compared to PBE and its hybrid derivatives (HSE06 and PBE0).

To gain further insight into the impact of the xc functional on the structural properties of the analyzed MOF-5 variants, we examine key interatomic distances, specifically the separation between the metal node (M) and its neighboring oxygen atoms (O1 and O2, see Figure 1b). Starting with pristine MOF-5 (Figure 3a, left), for which experimental data exists,^70^ we notice trends consistent with those obtained for the lattice parameter (Figure 2). In particular, the PBE functional predicts the longest M-O distances. Incorporating dispersion corrections shortens these bonds, particularly with the rVV10 method. The Grimme-D3 scheme has a negligible effect on the M-O1 distance and only a minor impact on M-O2. Hybrid functionals yield M-O1 separations close to those from PBE (Figure 3a, left), while M-O2 distances are more similar to those from R2SCAN (Figure 3c, left). The metaGGA approximation predicts the shortest bond lengths. Notably, the R2SCAN result (without dispersion correction) for the M-O2 distance matches the experimental value,^70^ as do PBE0 + Grimme-D3 (Figure 3c, left, and Table S3). For the M-O1 separation, R2SCAN (without dispersion correction) provides the best agreement with the experiment (Figure 3a, left) with a discrepancy of less than 0.01 Å. However, variations in bond lengths across different xc functionals and dispersion corrections are all within 0.03 Å with respect to each other and the experimental value,^70^ as testified by the RMSE computed for this quantity, see Table S4. In analogy to the trends obtained for the lattice parameter, PBE leads to the larger RMSEs of the order of 0.03 Å regardless of the dispersion correction. HSE06 gives rise to RMSEs ranging from 0.013 Å with rVV10 to 0.024 Å without vdW corrections. With the global hybrid PBE0, the RMSEs are further reduced to 0.012 Å with rVV10, 0.017 Å without dispersion correction, and 0.018 Å with Grimme-D3. Finally, the smallest RMSEs ≤ 0.01 Å are delivered by R2SCAN.

**Figure 3:**
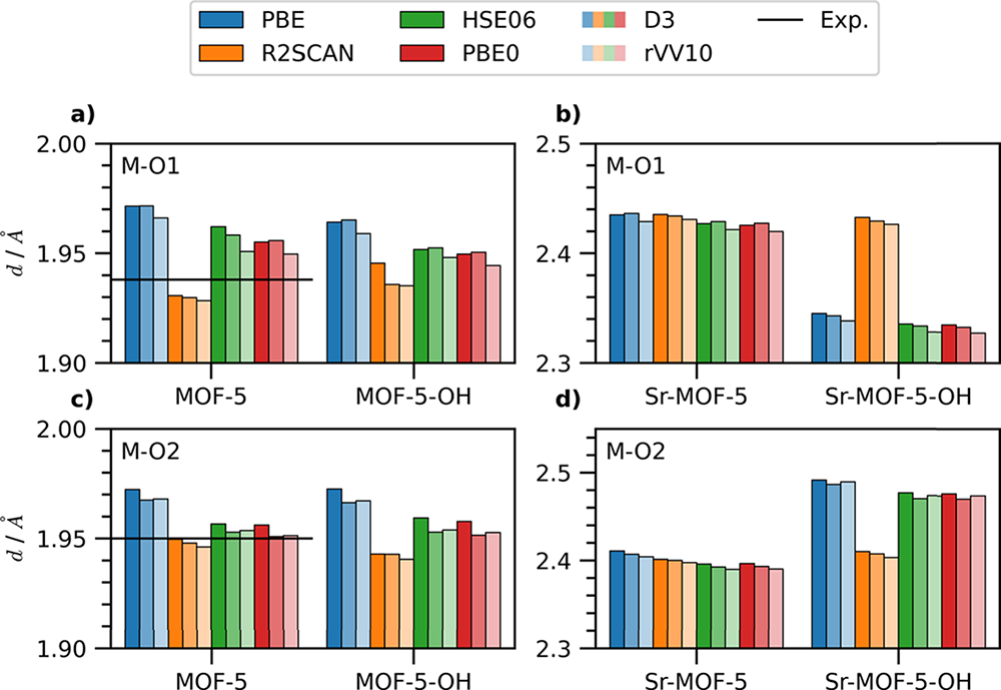
Interatomic distances between the metal atom and O1 (see Figure 1) in (a) conventional MOF-5 and its OH-functionalized counterpart, as well as in the (b) Sr-based variants with H- and OH-termination of the BDC linker. Interatomic distances between the metal atom and O2 (see Figure 1) in (c) conventional MOF-5 and its OH-functionalized counterpart, as well as in the (d) Sr-based variants with H- and OH-termination of the BDC linker. The experimental references available for conventional MOF-5 ^70^ are marked by a horizontal bar.

The trends obtained for the xc functionals and vdW treatments in pristine MOF-5 are mirrored in its hydroxyl-functionalized counterpart. PBE again predicts the largest interatomic distances, while R2SCAN yields the shortest. Hybrid functionals produce similar results, falling between those of PBE and R2SCAN (see Figure 3a,c, right). However, a subtle difference emerges regarding dispersion corrections: the Grimme-D3 correction with PBE, HSE06, and PBE0 results in larger M-O1 distances than the corresponding uncorrected functionals (Figure 3a, right). The same is obtained with R2SCAN for M-O2 (Figure 3c, right). Consistent with the results for pristine MOF-5, the variations in interatomic distances due to different xc functionals and vdW treatments remain on the order of 0.01 Å while the RMSE is slightly reduced with PBE, where the obtained values are around 0.02 Å and substantially lower for R2SCAN and the hybrid functionals where the RMSE is of the order of 10^–3^ Å or lower (Table S4).

The predicted interatomic distances for the H-passivated Sr-based MOF-5 variant agree with the calculated lattice parameter. Figure 3b and d (left panels) show the M–O bond lengths. PBE predicts the largest values, followed by R2SCAN and the hybrid functionals. Similar to MOF-5, dispersion corrections influence the bond lengths. The Grimme-D3 method increases M–O1 with all xc functionals except R2SCAN (Figure 3b). Conversely, the longest Sr–O2 distances are observed without dispersion corrections (Figure 3d). Including the rVV10 correction yields the shortest metal–oxygen separations for all tested approximations for *v*_xc_.

The structural analysis of OH-functionalized Sr-based MOF-5 reveals a more complex picture, although the calculated lattice parameters remain consistent with predictions from various xc functionals and vdW treatments. Unlike the other compounds examined so far, the Sr–O1 distance is approximately 0.1 Å shorter than the Sr–O2 distance, due to the significant distortion of the BDC molecule induced by concomitant Sr-substitution and hydroxyl functionalization.^19^ Consequently, the trends observed for Sr–O1 (Figure 3b, right) and Sr–O2 (Figure 3d, right) are mirrored. The structures calculated using R2SCAN, which are less distorted than those predicted by the other functionals, result in similar distances with and without linker functionalization. Further details are provided in Tables S1 and S2. On the other hand, the PBE functional without dispersion correction exhibits the largest value for the distance with Sr–O1. However, adding the Grimme-D3 correction to PBE decreases Sr–O1, in contrast with the effect obtained without it (Figure 3b, right). This same trend is achieved for the hybrid functionals with Grimme-D3, although in these cases, the Sr–O1 distance is shorter than that predicted by PBE. PBE0 and HSE06 yield nearly identical Sr–O1 separation, see Table S2. The rVV10 correction further decreases the distance. A similar pattern is obtained for Sr–O2, where only the rVV10 correction in combination with the hybrid functionals increases the distance.

### 3.2 Bader Charge Analysis

To gain insight into the charge distribution within the MOF variants, we perform Bader charge analysis. Although Bader charges are not physical observables and lack absolute reference values, we can analyze the trends obtained across different functionals and vdW corrections. For the H atoms passivating C3 in the BDC linker (Figure 4a), all adopted approaches predict small positive charges with a consistent trend: R2SCAN yields the largest values, followed by PBE0, HSE06, and PBE delivering similar results, see also Table S5. The inclusion of vdW corrections results in minor variations compared to the impact of the xc functional.

**Figure 4:**
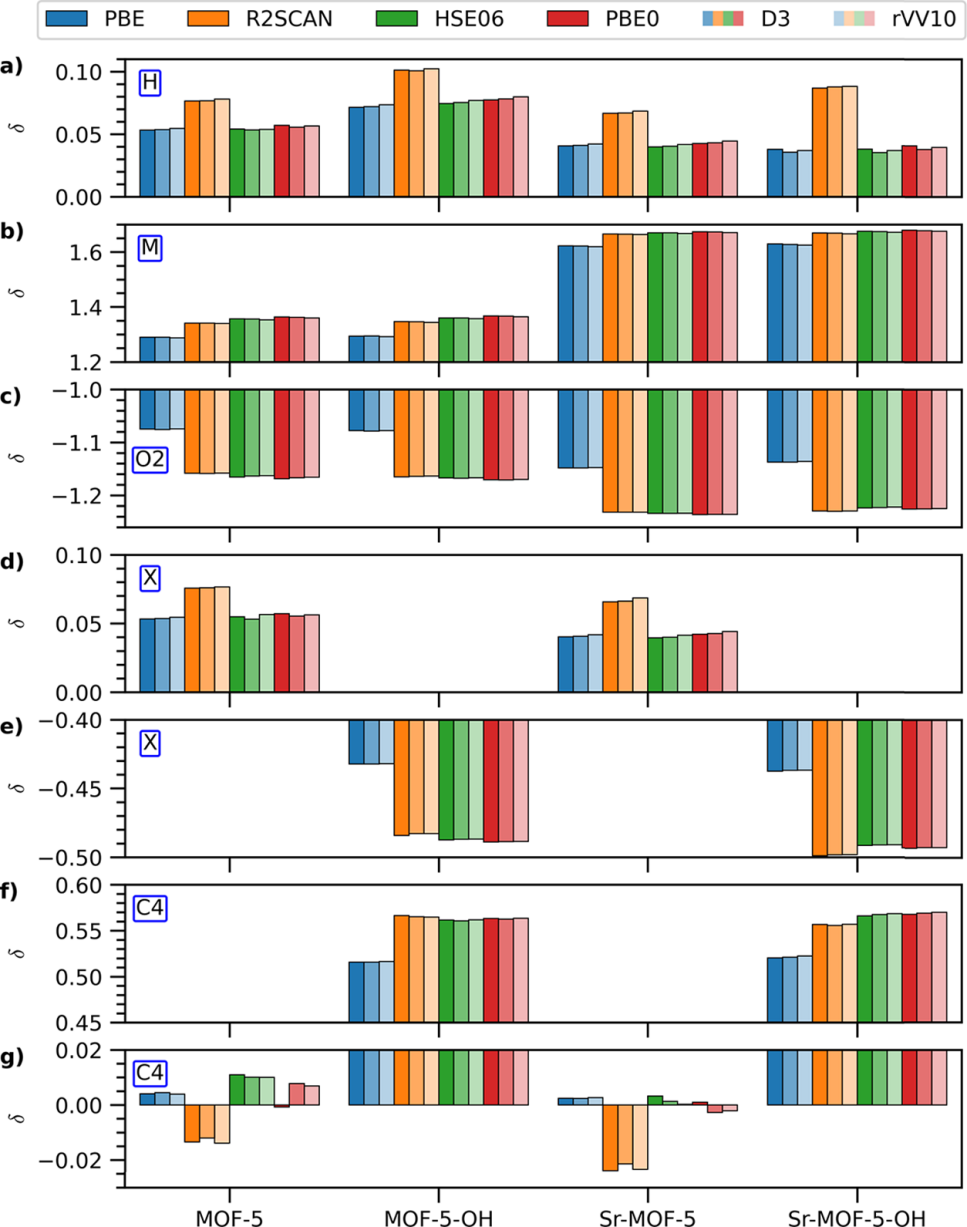
Bader charges with respect to the nominal atomic charges computed with different xc functionals and treatments of vdW interactions for (a) H atoms bound to C3 in the BDC, (b) on the metal node M, (c) on O2, (d) and (e) on the functional group X (H and OH), and (f) and (g) on the C atoms to which they are attached.

More significant differences emerge for the metal atoms (Figure 4b). The hybrid functionals PBE0 and HSE06 predict the largest positive charges on both Zn and Sr, likely due to the inclusion of a fraction of exact exchange which promotes charge localization. The semilocal PBE functional yields the smallest charges, while R2SCAN provides intermediate values close to the hybrids. vdW corrections have a negligible effect here (see Table S6). The consistently higher charges on Sr compared to Zn are attributed to the s-character of the outer shell of Sr.

The O2 oxygen atoms exhibit a significant accumulation of negative charge (Figure 4c), consistent with their electronegativity. The absolute value is larger in the Sr-based MOFs, mirroring the trend observed for the metal nodes. The divergence between PBE and higher-level functionals is notable here, with PBE0 predicting the most negative charges, with HSE06 and R2SCAN deviating from these values by a few thousandths of electrons (Table S7). This result highlights the known limitations of semilocal functionals in describing charge localization on electronegative atoms bonded to metals. Again, vdW corrections do not significantly alter these findings.

Distinct trends are obtained for the partial charges on the BDC functional group (H or OH, see Figure 4d,e). Positive charges are predicted on hydrogens with trends similar to those on the C3-bound atoms, and small variations due to vdW corrections (Table S8). Negative charges are found on OH groups, with semilocal PBE yielding the least negative values, while R2SCAN and the hybrid functionals produce similar, more negative charges. vdW corrections have a minimal impact on these results.

Finally, the charges on the carbon atom C4 anchoring the functional group reflect the electronegativity of the substituent (Figure 4f,g). In the H-passivated MOFs, the charges on C4 are near zero with some functional dependence: R2SCAN and HSE06 predict slightly larger values (≥0.01) compared to PBE and PBE0, which yield values below this threshold (Table S9). On the other hand, C4 atoms embedded in hydroxyl-functionalized MOFs exhibit positive charges, with PBE yielding the smallest and R2SCAN the largest values (though the latter are very similar to the results obtained with the hybrid functionals, see Table S9). The effect of vdW corrections on C4 charges varies depending on the functional and metal center. However, variations are on the order of 10^–3^ electrons in all cases.

In summary, our Bader charge analysis reveals notable trends related to the choice of the xc functional, particularly the tendency of hybrid functionals to predict more localized charges on metal and electronegative atoms, as expected. While vdW corrections generally have a smaller impact, the functional group significantly influences the charge distribution on the BDC linker. These observations provide valuable insights into the electronic structure of the investigated MOF variants.

### 3.3 Electronic Properties

We now inspect the electronic properties that are strongly influenced by the adopted xc functional, as expected. As shown in Figure 5(see also Table S10), the band gap predicted by PBE for conventional MOF-5 is 3.6 eV, while including a fraction of exact exchange increases it to 4.7 eV (HSE06) and 5.5 eV (PBE0). The result given by R2SCAN is between those,^42^ namely 3.9 eV. While the influence of vdW corrections on the band gap is 2 orders of magnitude smaller, careful inspection of Figure 5 reveals subtle differences. Excluding dispersion corrections or using the Grimme-D3 method results in slightly larger band gaps compared to the rVV10 scheme.

**Figure 5:**
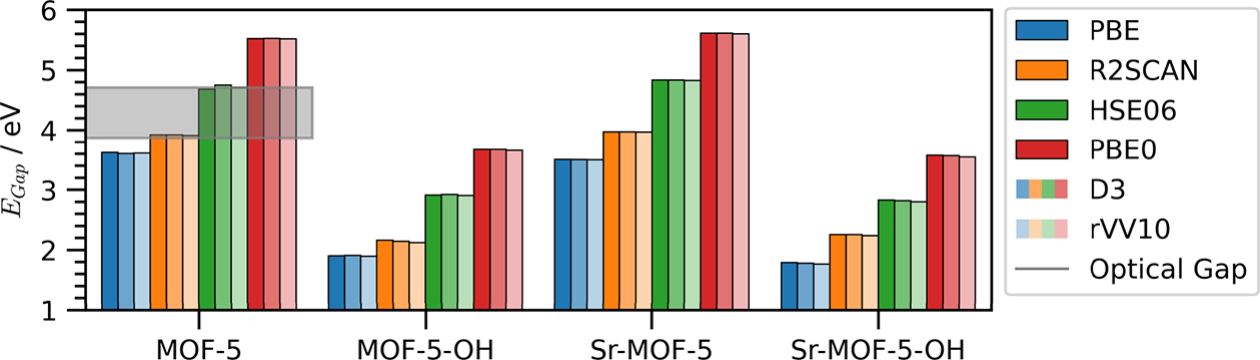
Fundamental gaps computed for conventional MOF-5, its Sr-substituted variant, and their OH-functionalized derivative using different xc functionals and vdW treatments. The range of available experimental values for the optical gaps of conventional MOF-5 ^72–74^ is indicated by a gray shaded rectangle.

In the absence of experimental references for electronic band gaps based on photoemission or transport measurements, which would validate the GW result reported in ref 75, we choose to contrast our DFT results against experimental optical absorption data for conventional MOF-5. Challenges related to this comparison have been extensively discussed in the literature.^74,76^ While DFT calculations notoriously neglect relevant physical effects, such as self-energy correction and electron–hole correlations, in MOFs, which combine a crystalline structure with organic building blocks, we expect that they largely cancel out,^75^ in analogy to purely organic crystals.^77^ Given the wide range of optical absorption data reported for MOF-5 ^72,73,78–80^ and the numerous approaches to process them,^74^ we take as a reference the window between 3.87 eV, extracted from a recent experimental study,^73^ and 4.71 eV, reported in ref 74 as a Gaussian fit of earlier optical absorption measurements.^72,73^ We emphasize that the following analysis cannot represent a rigorous validation of band gap calculations using different functionals. Nonetheless, it provides qualitative indications about the performance of the considered DFT approximations against a relevant (although indirectly related) experimental observable.

As shown in Figure 5, the R2SCAN and HSE06 results are within the above-mentioned experimental range, with the meta-GGA functional accurately reproducing the lower bound and the hybrid functional the upper one. The established ability of HSE06 to accurately predict optical gaps of solids,^42,81^ including MOFs,^74,82–84^ is well documented. The good performance of R2SCAN is particularly significant given the growing popularity of metaGGA functionals in MOF research^85,86^ and their demonstrated success in reproducing electronic gaps in technologically relevant materials.^42,87,88^ The performance of PBE is poor, although the values reported in Figure 5 are not dramatically below the range of experimental optical gaps due to the anticipated compensation between quasi-particle correction and exciton binding energy mentioned above. Conversely, PBE0 overestimates the optical gap because the fraction of Hartree–Fock exchange therein is not effectively screened, as in HSE06. For this reason, PBE0 is often favored for nonperiodic systems but less appropriate for crystalline solids.^89^

With the knowledge gained from this analysis of the band gap of MOF-5, we can continue with the inspection of its variants. While the trends obtained for pristine MOF-5, including the impact of dispersion corrections, are preserved regardless of the adopted xc functional (Figure 5), hydroxyl functionalization significantly reduces the gap.^19^ Specifically, the PBE gap is below 2 eV,^19^ R2SCAN yields approximately 2.2 eV, HSE06 results are close to 3 eV, and PBE0 predicts gaps of 3.8 eV. Substituting Zn with Sr does not substantially alter the gap: R2SCAN predicts a small increase, while PBE, HSE06, and PBE0 a slight decrease.

Next, we analyze the density of states (DOS) of conventional MOF-5 and its Sr-substituted OH-functionalized counterpart (Figure 6), focusing on how the different xc functionals reproduce the key features of the valence and conduction region. Given the minimal impact of different flavors of dispersion corrections, we present here only results obtained with the Grimme-D3 scheme. Further details are reported in Figures S1–S3. For conventional MOF-5, all xc functionals reproduce almost identically the prominent peaks in the occupied and unoccupied regions, see Figure 6a–d. No significant differences between the plots are visible, apart from the absolute energies of the conduction states, since all DOS are aligned to the valence band maximum. Subtle variations in the relative peak energies can be attributed to the specific approximations employed for *v*_xc_. For instance, in the unoccupied region, a prepeak at approximately 5.5 eV is present in the DOS calculated with PBE, PBE0, and HSE06, likely due to the semilocal treatment of correlation shared by these three functionals, as it is absent in the R2SCAN results (Figure 6b).

**Figure 6:**
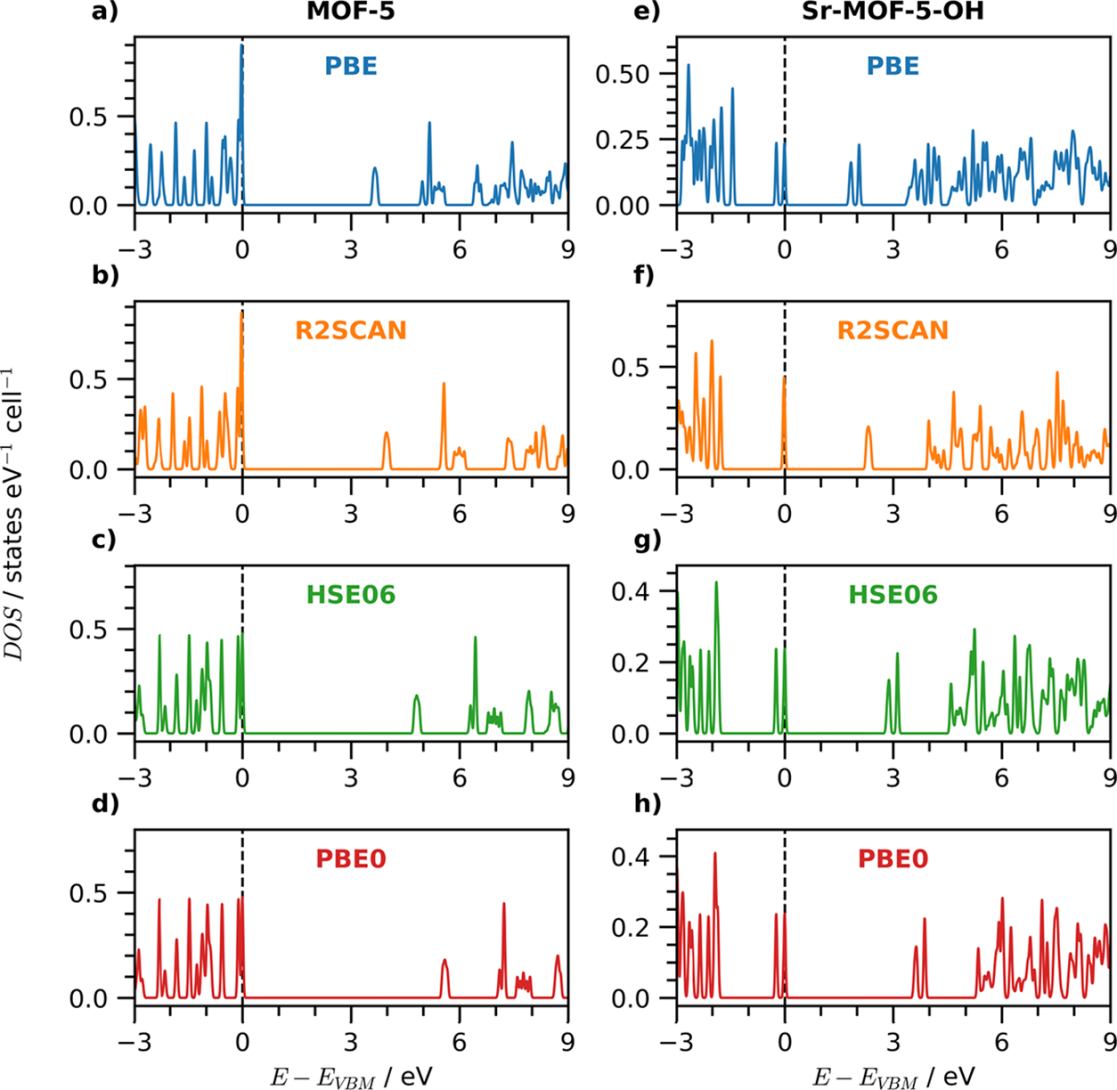
Density of states for conventional MOF-5 (left) computed using (a) PBE, (b) R2SCAN, (c) HSE06, and (d) PBE0, and its Sr-substituted hydroxyl functionalized counterpart (right) using (e) PBE, (f) R2SCAN, (g) HSE06, and (h) PBE0. In both cases, all functionals are supplemented with the Grimme-D3 method to deal with dispersion corrections. The energy scale is offset to the valence band maximum (VBM) set to zero.

The electronic structure of MOF-5 remains substantially unaltered in its Sr-substituted OH-functionalized variant, although some discrepancies arise depending on the adopted xc functional (Figure 6e–h). The PBE results show a dominant, sharp peak in the valence region around −2.5 eV (Figure 6d). This peak shifts to lower energies with both HSE06 and PBE0 (Figure 6g,h) while it is not featured by R2SCAN (Figure 6f), which, on the other hand, yields a different description of the highest occupied valence states compared to the other xc functionals. The most striking variation is the presence of only one peak both at the top of the valence band and at the bottom of the conduction band, in contrast to PBE, HSE06 and PBE0 which predict two peaks in both cases. The origin of this splitting was attributed to structural distortions,^19^ which promote hybridization between the functional group and BDC as predicted by PBE, HSE06, and PBE0 calculations.

### 3.4 Phonon Dispersion

In the last part of our analysis, we investigate the phonon band structure computed with PBE, R2SCAN, and PBE0 in conjunction with the Grimme-D3 and rVV10 schemes for dispersion corrections. Due to the higher computational costs, we considered only one hybrid functional (PBE0) with looser settings compared to PBE and R2SCAN (see Section 2.2). Furthermore, we focused only on conventional MOF-5, as exploring BDC functionalization increases the complexity of the potential energy surface, thus dramatically complicating the search for the structural minimum of the material.

No imaginary frequencies appear in the phonon band structure obtained with the PBE functional (Figure 7a), indicating the dynamic stability of MOF-5 and the ability of this approximation to describe it. This finding is robust even in the absence of dispersion corrections, with the inclusion of the Grimme-D3 or rVV10 inducing only minor modifications in the phonon spectra (Figure 7a–c) that are hardly discernible from visual inspection. Calculations performed with R2SCAN, both without dispersion corrections and with the Grimme-D3 correction, yield phonon frequencies closely matching those from PBE. Slightly softer modes in the low-frequency region can be attributed to the higher cutoff employed (see Section 2.2). In contrast, R2SCAN with vdW corrections using the rVV10 scheme feature imaginary modes with frequencies of −0.3 THz, indicative of slight dynamic instabilities, when the standard computational settings reported in Section 2.2 are employed. By reducing the force threshold by 2 orders of magnitude (1.9 × 10^–7^ Ha/bohr), the correct structural minimum can be achieved with all modes being real, see Figure 7f.

**Figure 7:**
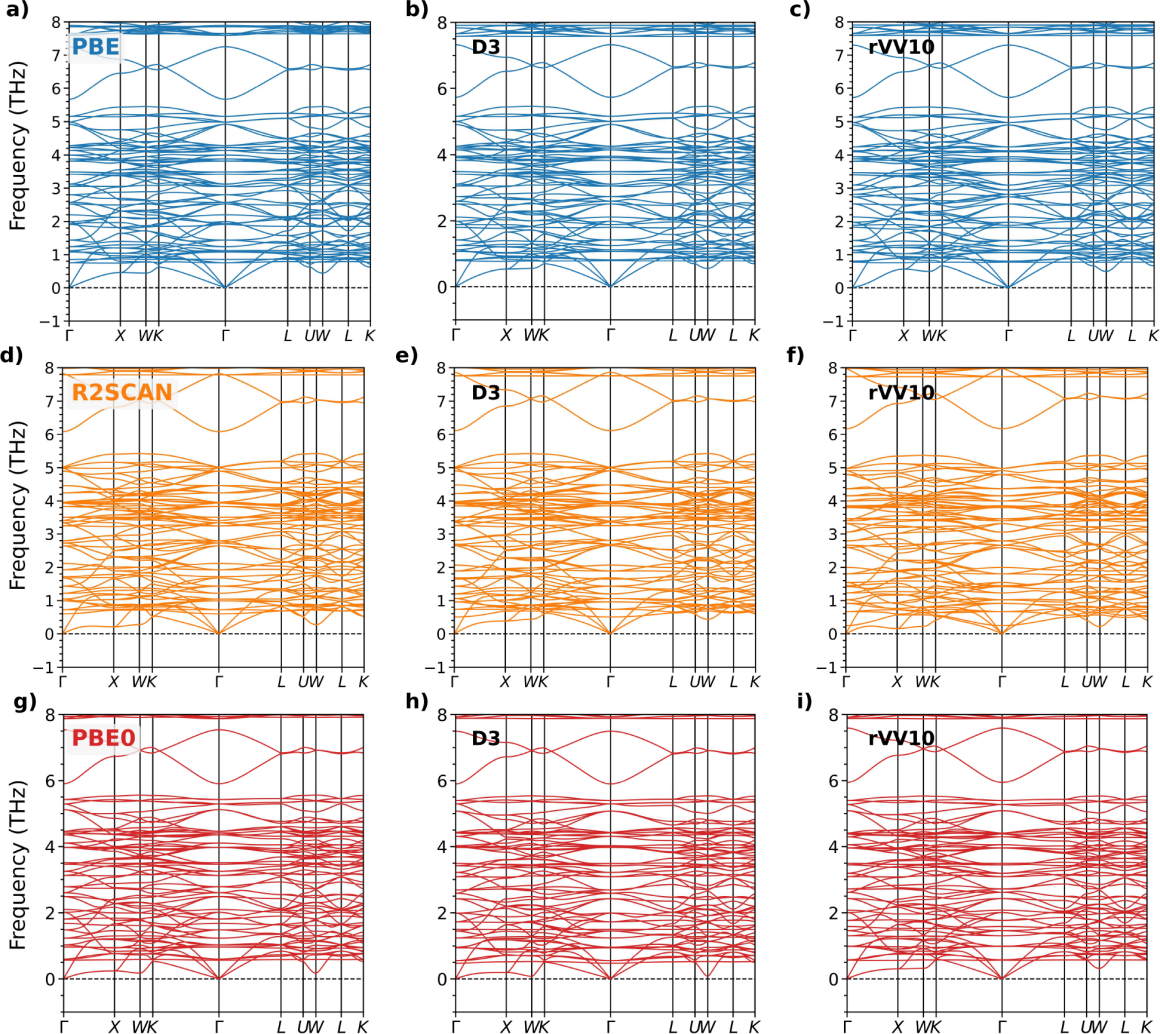
Phonon dispersion curves of primitive MOF-5 calculated with PBE (a) without dispersion corrections, (b) with the Grimme-D3 method, and (c) with rVV10; with R2SCAN (d) without dispersion corrections, (e) with the Grimme-D3 method, and (f) with rVV10; and with PBE0 (g) without dispersion corrections, (h) with the Grimme-D3 method, and (i) with rVV10.

The hybrid functional PBE0 yields phonon spectra generally similar to those given by R2SCAN, especially when no vdW corrections are applied (Figure 7g). The effect of the Grimme-D3 method on top of the PBE0 result is very minor compared to the other xc functionals (Figure 7h). In particular, the low-frequency modes around W approach zero in contrast to the outcomes of phonon calculations with PBE + D3 and R2SCAN + D3 (compare Figure 7b,e,h). Using the rVV10 scheme on top of PBE0 delivers a phonon spectrum closely resembling the other two without vdW corrections and with D3, see Figure 7i. The main differences lie again in the lowest modes around W, which appear at higher frequencies compared to the other approaches. Overall, the softer phonon modes predicted by PBE0 can be ascribed to two main factors: (i) the use of a smaller (1 × 1 × 1) supercell, which truncates long-range interatomic interactions, especially affecting low-frequency acoustic modes, and (ii) the inclusion of 25% Hartree–Fock exchange which alters the interatomic force constants, thus affecting bond strengths and vibrational properties.^90^

We finally computed the phonon dispersion of the Sr-substituted MOF-5 using the PBE, R2SCAN, and PBE0 functionals supplemented only by the Grimme-D3 scheme for vdW corrections, see Figure 8. This material is predicted to be dynamically stable by all considered functionals, a relevant result for stimulating the synthesis of this compound. The harder modes predicted by R2SCAN (Figure 8b) compared to PBE (Figure 8a) and PBE0 (Figure 8c) can be attributed to the tighter lattice parameters predicted by this functional. In turn, PBE0 predicts higher phonon frequencies compared to PBE for the same reasons discussed above for conventional MOF-5. Comparing Figure 8 with Figure 7, we notice that the substitution of Zn with Sr leads to an overall softening of the phonon modes, which can be primarily ascribed to the higher atomic mass of Sr compared to Zn.

**Figure 8:**
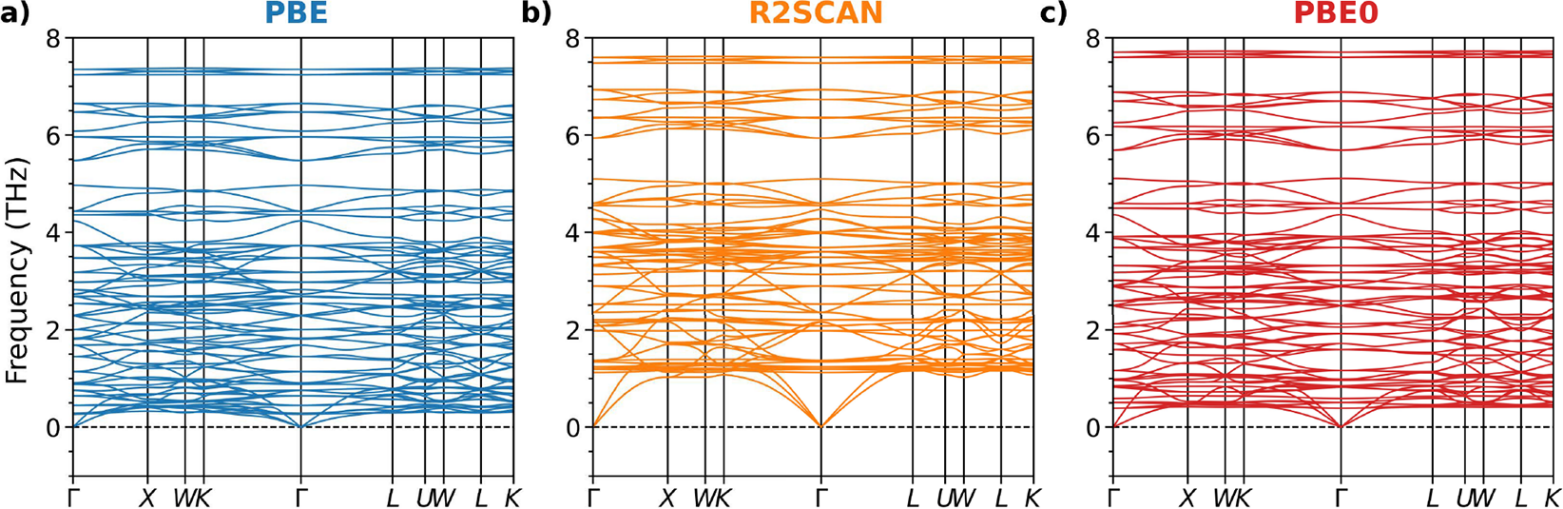
Phonon dispersion curves of Sr-substituted MOF-5 computed with (a) PBE, (b) R2SCAN, and (c) PBE0, all supplemented by the Grimme-D3 method for treating vdW interactions.

Our results indicate that R2SCAN is the most effective method for calculating phonon dispersion in MOF-5 and its variants. Its accurate prediction of low-frequency modes, consistent with inelastic neutron scattering data,^91^ can be ascribed to its robust treatment of intermediate- and long-range interactions, as discussed in previous studies.^92–95^ While PBE0 also captures these modes, its higher computational cost is a significant drawback. Dispersion corrections preserve the dynamical stability of MOF-5 while improving the prediction of its structural properties, see Section 3.1. However, the computational costs associated with rVV10 make R2SCAN + D3 the optimal choice for reliable and efficient phonon dispersion calculations of MOF-5.

## 4 Discussion

The results presented in this study offer valuable guidance for choosing the most appropriate xc functional and dispersion corrections for MOF-5 and its derivatives, considering both accuracy and computational costs (Figure 9). For lattice parameters, R2SCAN + D3 provides excellent predictions (Figure 2) with similar trends obtained for interatomic distances (Figure 3). While hybrid functionals HSE06 and PBE0, both with Grimme-D3, achieve comparable accuracy for H-terminated MOF-5 variants, they are significantly more computationally expensive, requiring approximately ten times the runtime (Figure 9a and Table S11). Optimizing hydroxyl-functionalized structures is generally more demanding. For OH-terminated MOF-5 (Zn metal node), R2SCAN + D3 remains the optimal trade-off between accuracy and cost, though the advantage over hybrid functionals is reduced to approximately a factor of 5, see Figure 9a. For the hydroxyl-functionalized Sr-substituted MOF-5, the hybrid functionals are as expensive as R2SCAN and PBE, which, in turn, require almost 10 times more core hours than for the H-passivated counterpart. We can attribute this behavior to the considerable distortions in this system caused by the larger atomic radius of Sr compared to Zn and the presence of the OH group, which distorts the organic scaffold. These combined effects make structural optimization considerably more demanding than for the other systems examined in this work. We have to point out that the structures predicted with the hybrid functionals use the results from PBE as input.

**Figure 9:**
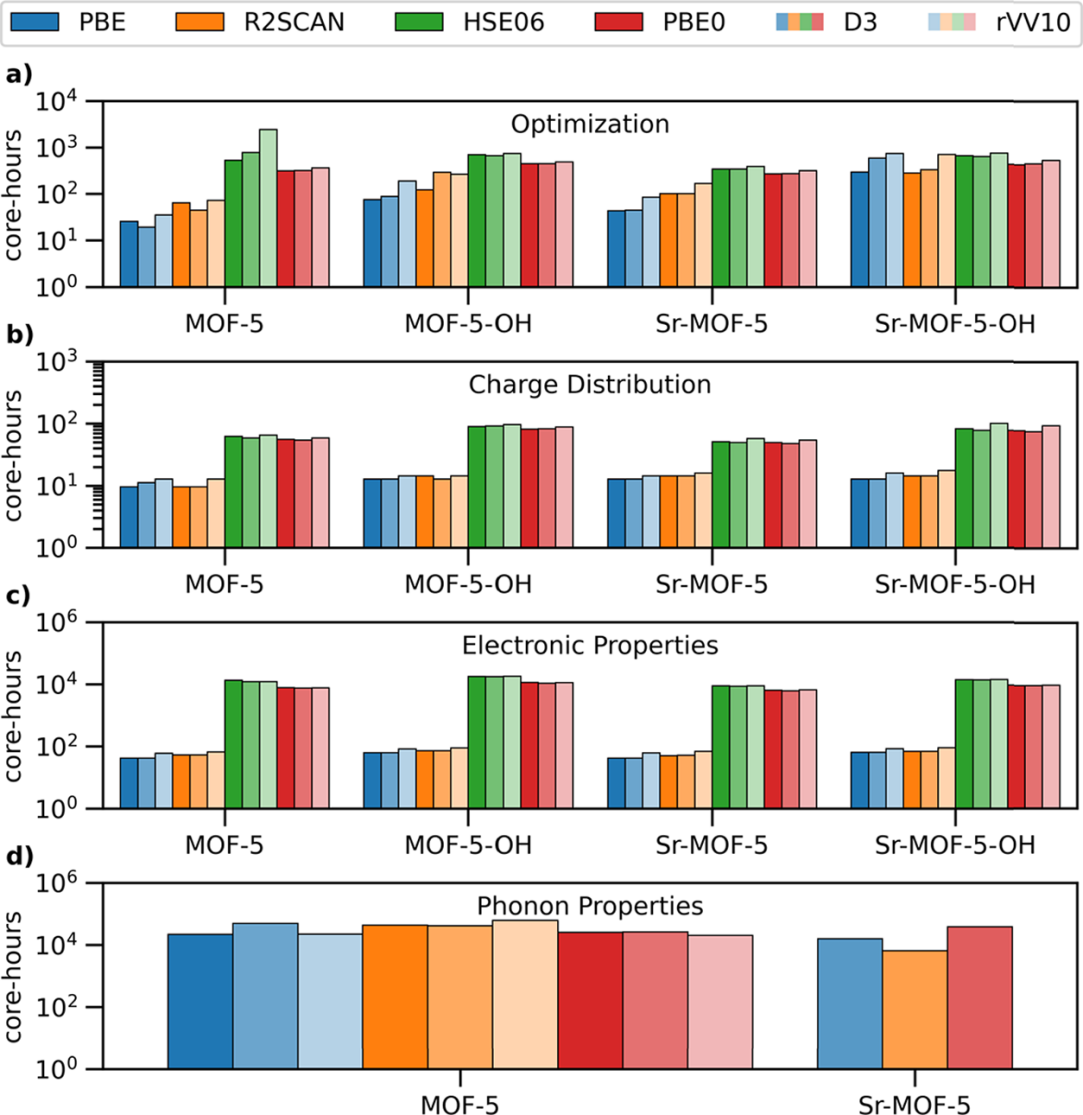
Core-hours required for (a) structural optimization, (b) Bader charges, (c) electronic properties, and (d) phonon properties of MOF-5 and derivatives using different xc functionals and dispersion corrections.

For the Bader charges, HSE06 + rVV10 provided the best agreement between our predictions and experimental references for conventional MOF-5 (Figure 4). However, this method is computationally expensive, see Figure 9b and Table S12. Since different xc functionals and dispersion corrections affect Bader charges only by hundredths of an electron, the R2SCAN + D3 method is the most viable alternative for both Zn-based MOF-5 (with and without OH termination) and its Sr-substituted variants.

In contrast, electronic-structure calculations of MOFs are very sensitive to the choice of the xc functional, both in terms of accuracy and computational costs. As illustrated in Figure 9c (see also Table S13), hybrid functionals require 2 orders of magnitude more core hours than PBE or R2SCAN, regardless of the system. Similarly, the rVV10 method for treating vdW interactions is significantly more expansive than Grimme-D3. Considering these factors, and the band gap results presented in Figure 5, R2SCAN + D3 offers again the best balance between accuracy and computational efficiency.

Finally, the calculation of phonon dispersions is very expensive even with the PBE functional, see Figure 9d and Table S14. The inclusion of vdW corrections, especially with the Grimme-D3 method, further increments the computational costs. Those imposed by R2SCAN are a factor 2 larger than those of PBE, while PBE0 results, obtained on a single unit cell with 106 atoms (see Section 2.2) are comparable with those obtained with the semilocal functional. For Sr-substituted MOF-5, R2SCAN + D3 calculations are cheaper than PBE + D3 and PBE0 + D3, see Figure 9d, confirming that R2SCAN + D3 is the most suitable approach for calculating the phonon properties of MOF-5 and its variants.

## 5 Summary and Conclusions

In summary, we benchmarked the performance of four exchange-correlation functionals (PBE, R2SCAN, HSE06, and PBE0) and two dispersion correction schemes (Grimme-D3 and rVV10) on the structural, electronic, and vibrational properties of MOF-5 and its Sr-substituted and/or hydroxyl-functionalized derivatives using DFT. R2SCAN + D3 emerged as the optimal choice for calculating structural properties, accurately predicting lattice parameters and interatomic distances and requiring about ten times lower runtime than the hybrid functionals. Regarding Bader charges, while HSE06 + rVV10 provided the best agreement with experimental data for MOF-5, its high computational cost makes R2SCAN + D3 a cheaper alternative for comparable accuracy. Electronic structure calculations proved to be highly sensitive to the approximation of *v*_xc_, as expected. Hybrid functionals demand 2 orders of magnitude more computational resources than PBE or R2SCAN, without a commensurate improvement in accuracy. Similarly, the rVV10 dispersion correction is significantly more expensive than the Grimme-D3 scheme. Considering these factors, R2SCAN + D3 provides again the best compromise between accuracy and computational efficiency. Phonon calculations, even with PBE, are computationally demanding, with the inclusion of dispersion corrections further increasing their costs. While R2SCAN calculations are more expensive than PBE by a factor of 2, the choice of the vdW treatment does not significantly affect this trend. Importantly, for the Sr-substituted MOF-5, R2SCAN + D3 is surprisingly less expensive than PBE + D3. This finding, along with the accuracy observed for other properties, confirms R2SCAN + D3 as the most suitable functional for calculating phonon properties in MOF-5 and its variants.

In conclusion, our work demonstrates that R2SCAN + D3 offers an excellent balance between accuracy and computational cost for predicting the intrinsic properties of MOF-5 and its derivatives. Our results provide valuable guidance for future computational studies on MOFs and related materials, enabling efficient and reliable predictions of key properties in high-throughput calculations, where more advanced calculations, based, for example, on many-body perturbation theory,^75^ are prohibitive. As such, our study paves the way for accelerated computational design of MOFs with enhanced properties for applications in gas sensing and storage, which require simultaneously accurate predictions of structural, electronic, and vibrational properties.

## Supplementary Material



## Data Availability

The data produced in this study are openly available in Zenodo at DOI: 10.5281/zenodo.15005082.

## Notes

Additional information regarding structural and electronic properties and computational costs (PDF)
