# Supplementary material for: Benchmarking Selected Density Functionals and Dispersion Corrections for MOF‑5 and Its Derivatives
Source: J Chem Theory Comput. 2025 Jul 14;21(14):7062–74. doi: 10.1021/acs.jctc.5c00399 (PMC12288010; doi:10.1021/acs.jctc.5c00399)
Supplement: Supplementary file 1 [file ct5c00399_si_001.pdf]

SUPPORTING INFORMATION
Benchmarking Selected Density Functionals and Dispersion Corrections for MOF-5 and its Derivatives

Joshua Edzards,*,† Julia Santana Andreo,† Holger-Dietrich Saßnick,† and Caterina Cocchi*,†

†*Carl von Ossietzky Universität Oldenburg, Institute of Physics, 26129 Oldenburg, Germany*

‡*Friedrich-Schiller Universität Jena, Institute for Condensed Matter Theory and Optics, 07743 Jena, Germany*

E-mail: joshua.edzards@uni-oldenburg.de; caterina.cocchi@uni-jena.de

S1 Structural Properties

Table S1: Lattice parameter a in Å of the systems considered in this work computed with different functionals and dispersion corrections.

Functional	Dispersion Correction	MOF-5	MOF-5-OH	Sr-MOF-5	Sr-MOF-5-OH
	without	26.133	26.171	28.678	27.392
PBE	D3	26.098	26.131	28.655	27.277
	rVV10	26.091	26.123	28.630	27.247
	without	25.891	25.932	28.535	28.603
R2SCAN	D3	25.881	25.913	28.525	28.581
	rVV10	25.865	25.894	28.503	28.555
	without	25.937	25.980	28.474	27.235
HSE06	D3	25.899	25.943	28.455	27.108
	rVV10	25.890	25.935	28.430	27.093
	without	25.921	25.965	28.465	27.233
PBE0	D3	25.888	25.928	28.445	27.104
	rVV10	25.874	25.921	28.420	27.089

Table S2: Distances d in Å between the metal atom and O1 in the systems considered in this work computed with different functionals and dispersion corrections.

Functional	Dispersion Correction	MOF-5	MOF-5-OH	Sr-MOF-5	Sr-MOF-5-OH
	without	1.971	1.964	2.435	2.345
PBE	D3	1.972	1.965	2.436	2.343
	rVV10	1.966	1.959	2.429	2.338
	without	1.931	1.946	2.435	2.433
R2SCAN	D3	1.930	1.936	2.434	2.429
	rVV10	1.928	1.935	2.431	2.426
	without	1.962	1.952	2.427	2.335
HSE06	D3	1.958	1.952	2.429	2.333
	rVV10	1.951	1.948	2.422	2.328
	without	1.955	1.950	2.425	2.335
PBE0	D3	1.956	1.950	2.427	2.332
	rVV10	1.950	1.944	2.420	2.327

Table S3: Distances d in Å between the metal atom and O2 in the systems considered in this work computed with different functionals and dispersion corrections.

Functional	Dispersion Correction	MOF-5	MOF-5-OH	Sr-MOF-5	Sr-MOF-5-OH
PBE	without	1.972	1.972	2.411	2.491
	D3	1.967	1.966	2.407	2.486
	rVV10	1.968	1.967	2.404	2.466
R2SCAN	without	1.950	1.943	2.401	2.410
	D3	1.948	1.943	2.400	2.407
	rVV10	1.946	1.940	2.397	2.403
HSE06	without	1.957	1.959	2.396	2.477
	D3	1.953	1.953	2.393	2.470
	rVV10	1.954	1.954	2.390	2.474
PBE0	without	1.956	1.958	2.396	2.476
	D3	1.951	1.951	2.393	2.470
	rVV10	1.951	1.953	2.390	2.474

Table S4: Root mean square error (RMSE) in Å for conventional MOF-5 defined as $\sqrt{\frac{\sum(calc-exp)^2}{N}}$, where $calc$ represents the calculated value for the lattice parameter a, the distance between the metal atom and O1 (d_{M-O1}), and the distance between the metal atom and O2 (d_{M-O2}), exp is the experimental reference value from Ref. 1, and N is the number of values used for each quantity: $N = 3$ for a, $N = 8$ for d_{M-O1}, and $N = 24$ for d_{M-O2}.

Functional	Dispersion Correction	RMSE(a)	RMSE(d_{M-O1})	RMSE(d_{M-O2})
PBE	without	0.253	0.033	0.021
	D3	0.218	0.034	0.017
	rVV10	0.211	0.028	0.018
R2SCAN	without	0.011	0.007	0.001
	D3	0.001	0.008	0.002
	rVV10	0.015	0.010	0.004
HSE06	without	0.057	0.025	0.011
	D3	0.019	0.020	0.003
	rVV10	0.015	0.013	0.007
PBE0	without	0.041	0.017	0.006
	D3	0.008	0.018	0.001
	rVV10	0.006	0.012	0.001

S2 Bader Charges

Table S5: Bader charges on the H atoms on the linker bound to C3 computed with the Bader scheme using different functionals and dispersion corrections.

Functional	Dispersion Correction	MOF-5	MOF-5-OH	Sr-MOF-5	Sr-MOF-5-OH
	without	0.053	0.072	0.041	0.038
PBE	D3	0.054	0.072	0.041	0.036
	rVV10	0.055	0.074	0.042	0.037
	without	0.077	0.101	0.067	0.087
R2SCAN	D3	0.077	0.101	0.067	0.088
	rVV10	0.078	0.102	0.068	0.088
	without	0.054	0.075	0.040	0.038
HSE06	D3	0.053	0.075	0.040	0.035
	rVV10	0.054	0.077	0.042	0.037
	without	0.057	0.077	0.043	0.041
PBE0	D3	0.056	0.078	0.043	0.038
	rVV10	0.057	0.080	0.045	0.040

Table S6: Bader charges on the metal atoms computed with the Bader scheme using different functionals and dispersion corrections.

Functional	Dispersion Correction	MOF-5	MOF-5-OH	Sr-MOF-5	Sr-MOF-5-OH
	without	1.290	1.294	1.622	1.629
PBE	D3	1.290	1.294	1.622	1.628
	rVV10	1.288	1.292	1.619	1.625
	without	1.341	1.347	1.666	1.669
R2SCAN	D3	1.341	1.346	1.665	1.669
	rVV10	1.340	1.344	1.663	1.666
	without	1.357	1.360	1.670	1.676
HSE06	D3	1.356	1.360	1.670	1.674
	rVV10	1.353	1.357	1.667	1.672
	without	1.363	1.367	1.673	1.679
PBE0	D3	1.363	1.367	1.673	1.677
	rVV10	1.360	1.364	1.670	1.675

Table S7: Bader charges on the O2 atoms computed with the Bader scheme using different functionals and dispersion corrections.

Functional	Dispersion Correction	MOF-5	MOF-5-OH	Sr-MOF-5	Sr-MOF-5-OH
PBE	without	-1.075	-1.078	-1.148	-1.137
	D3	-1.076	-1.079	-1.148	-1.137
	rVV10	-1.075	-1.078	-1.148	-1.136
R2SCAN	without	-1.158	-1.165	-1.232	-1.229
	D3	-1.159	-1.164	-1.232	-1.230
	rVV10	-1.158	-1.163	-1.231	-1.229
HSE06	without	-1.165	-1.167	-1.234	-1.223
	D3	-1.163	-1.168	-1.234	-1.223
	rVV10	-1.163	-1.167	-1.234	-1.222
PBE0	without	-1.169	-1.171	-1.236	-1.226
	D3	-1.167	-1.171	-1.236	-1.225
	rVV10	-1.166	-1.170	-1.236	-1.225

Table S8: Bader charges on the functional groups H or OH computed with the Bader scheme using different functionals and dispersion corrections.

Functional	Dispersion Correction	MOF-5	MOF-5-OH	Sr-MOF-5	Sr-MOF-5-OH
PBE	without	0.053	-0.432	0.040	-0.437
	D3	0.054	-0.432	0.041	-0.437
	rVV10	0.055	-0.432	0.042	-0.437
R2SCAN	without	0.076	-0.484	0.066	-0.499
	D3	0.076	-0.483	0.066	-0.498
	rVV10	0.077	-0.483	0.069	-0.498
HSE06	without	0.055	-0.487	0.040	-0.491
	D3	0.053	-0.487	0.040	-0.491
	rVV10	0.056	-0.487	0.041	-0.491
PBE0	without	0.057	-0.489	0.042	-0.493
	D3	0.055	-0.489	0.043	-0.493
	rVV10	0.056	-0.489	0.044	-0.493

Table S9: Bader charges on the C4 atoms computed with the Bader scheme using different functionals and dispersion corrections.

Functional	Dispersion Correction	MOF-5	MOF-5-OH	Sr-MOF-5	Sr-MOF-5-OH
PBE	without	0.004	0.516	0.002	0.520
	D3	0.004	0.516	0.002	0.521
	rVV10	0.004	0.517	0.003	0.523
R2SCAN	without	-0.013	0.566	-0.024	0.557
	D3	-0.012	0.565	-0.021	0.556
	rVV10	-0.014	0.565	-0.023	0.557
HSE06	without	0.011	0.562	0.003	0.566
	D3	0.010	0.561	0.001	0.568
	rVV10	0.010	0.562	0.000	0.568
PBE0	without	-0.001	0.563	0.001	0.568
	D3	0.008	0.562	-0.003	0.569
	rVV10	0.007	0.564	-0.002	0.570

S3 Electronic Properties

Table S10: Fundamental gaps in eV computed for the systems considered in this work using different functionals and dispersion corrections.

Functional	Dispersion Correction	MOF-5	MOF-5-OH	Sr-MOF-5	Sr-MOF-5-OH
PBE	without	3.621	1.903	3.505	1.791
	D3	3.604	1.907	3.503	1.778
	rVV10	3.609	1.895	3.499	1.765
R2SCAN	without	3.922	2.162	3.971	2.253
	D3	3.922	2.143	3.971	2.253
	rVV10	3.910	2.122	3.967	2.238
HSE06	without	4.682	2.912	4.834	2.826
	D3	4.750	2.921	4.835	2.816
	rVV10	4.709	2.902	4.826	2.799
PBE0	without	5.520	3.672	5.608	3.576
	D3	5.523	3.673	5.610	3.569
	rVV10	5.515	3.660	5.600	3.547

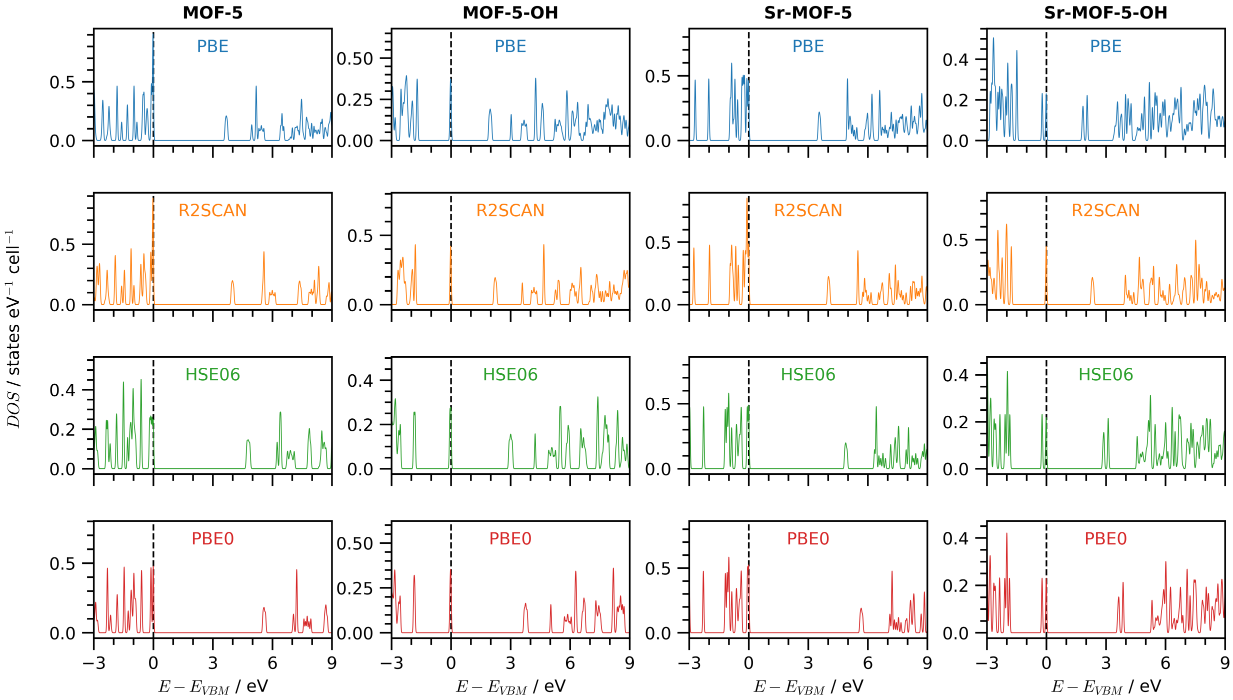

Figure S1: Density of states computed for the systems considered in this work using different functionals without dispersion corrections. The energy scale is set to zero at the valence band maximum (VBM).

S4 Computing Time

Table S11: Core-hours required for the structural optimization of MOF-5 and derivatives using different functionals and dispersion corrections.

Functional	Dispersion Correction	MOF-5	MOF-5-OH	Sr-MOF-5	Sr-MOF-5-OH
PBE	without	25.6	75.2	43.2	292.8
	D3	19.2	88.0	44.8	593.6
	rVV10	35.2	188.8	84.8	745.6
R2SCAN	without	64.0	121.6	100.8	278.4
	D3	44.8	289.6	1.8	331.2
	rVV10	72.0	262.4	168.0	710.4
HSE06	without	529.6	699.2	340.8	664.0
	D3	779.2	665.6	339.2	640.0
	rVV10	2433.6	744.0	385.6	761.6
PBE0	without	312.0	446.4	268.8	432.0
	D3	316.8	446.4	270.4	452.8
	rVV10	363.2	488.0	313.6	534.4

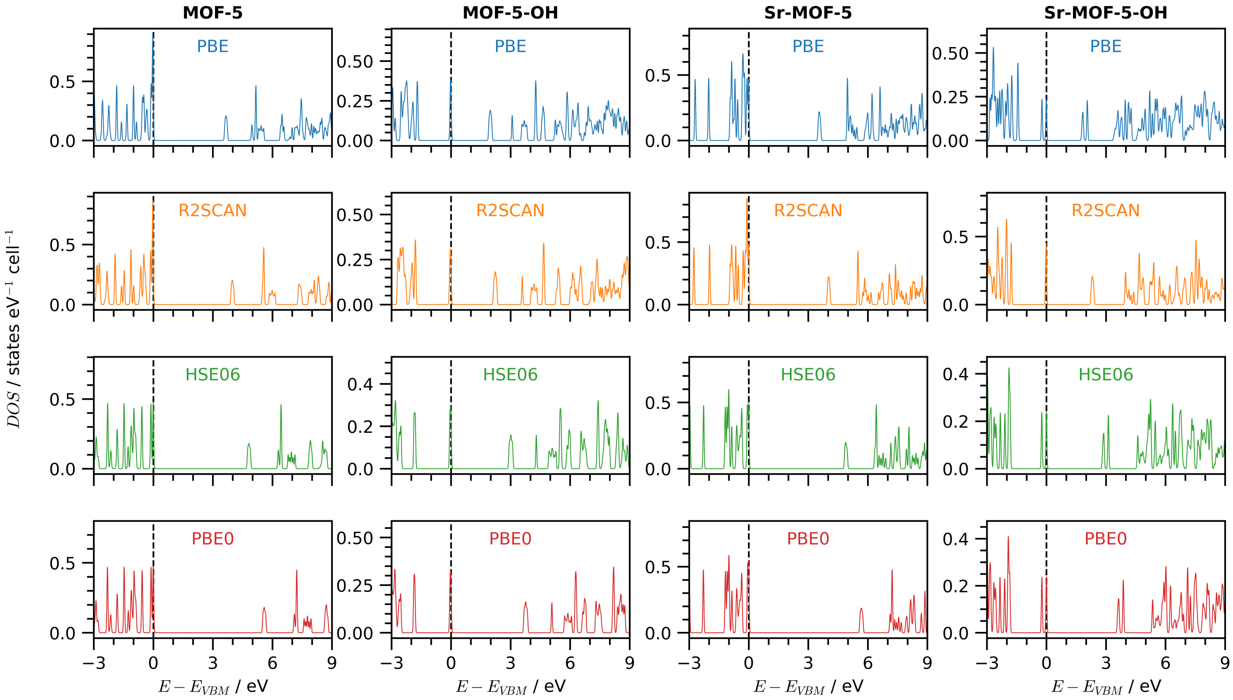

Figure S2: Density of states computed for the systems considered in this work using different functionals and the Grimme-D3 scheme for dispersion corrections. The energy scale is set to zero at the valence band maximum (VBM).

Table S12: Core-hours required for the calculation of Bader charges of MOF-5 and derivatives using different functionals and dispersion corrections.

Functional	Dispersion Correction	MOF-5	MOF-5-OH	Sr-MOF-5	Sr-MOF-5-OH
PBE	without	9.6	12.8	12.8	12.8
	D3	11.2	12.8	12.8	12.8
	rVV10	12.8	14.4	14.4	16.0
R2SCAN	without	9.6	14.4	14.4	14.4
	D3	9.6	12.8	14.4	14.4
	rVV10	12.8	14.4	16.0	17.6
HSE06	without	62.4	89.6	51.2	83.2
	D3	59.2	91.2	49.6	78.4
	rVV10	65.6	96.0	57.6	1.8
PBE0	without	56.0	81.6	49.6	78.4
	D3	54.4	83.2	48.0	75.2
	rVV10	59.2	88.0	54.4	94.4

References

(1) Li, H.; Eddaoudi, M.; O'Keeffe, M.; Yaghi, O. M. Design and synthesis of an exceptionally stable and highly porous metal-organic framework. *Nature* **1999**, *402*, 276–279.

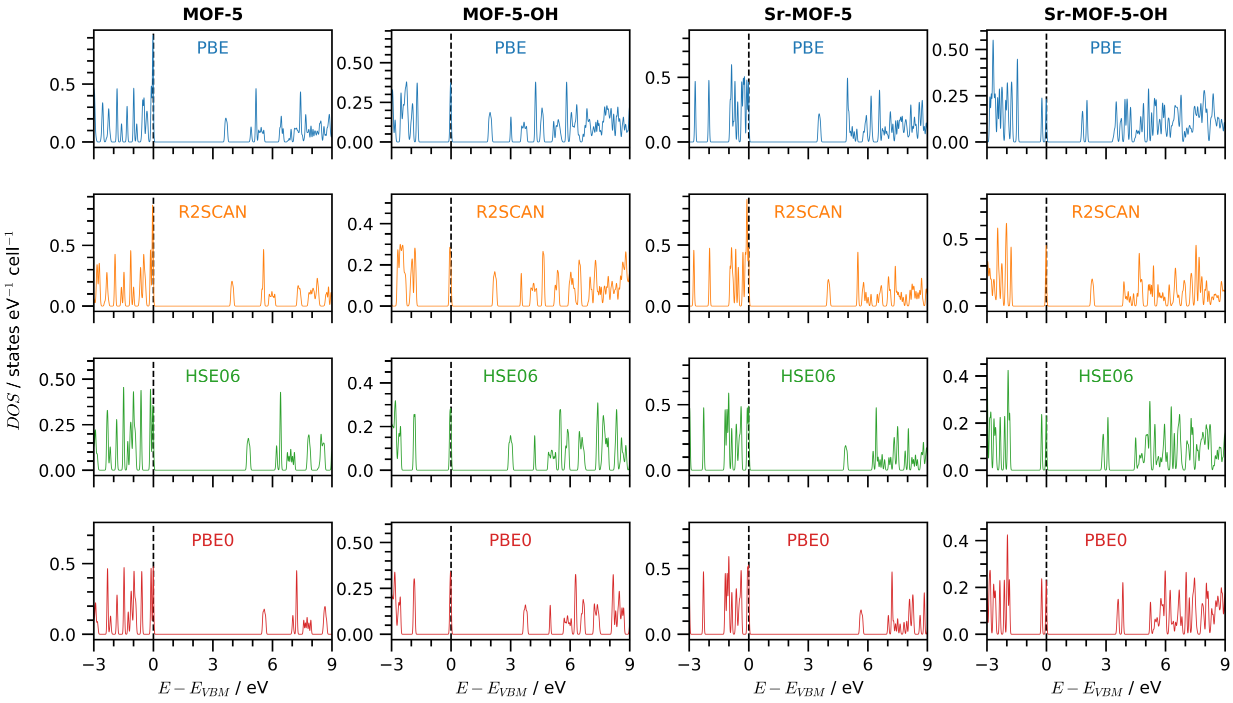

Figure S3: Density of states computed for the systems considered in this work using different functionals and the rVV10 scheme for dispersion corrections. The energy scale is set to zero at the valence band maximum (VBM).

Table S13: Core-hours required for the calculation of electronic properties of MOF-5 and derivatives using different functionals and dispersion corrections.

Functional	Dispersion Correction	MOF-5	MOF-5-OH	Sr-MOF-5	Sr-MOF-5-OH
PBE	without	41.6	62.4	41.6	64.0
	D3	41.6	62.4	41.6	64.0
	rVV10	59.2	81.6	60.8	83.2
R2SCAN	without	52.8	72.0	49.6	68.8
	D3	52.8	72.0	51.2	68.8
	rVV10	65.6	88.0	68.8	89.6
HSE06	without	13414.4	17766.4	8832.0	14003.2
	D3	12032.0	17382.4	8576.0	13900.8
	rVV10	12057.6	17945.6	8883.2	14208.0
PBE0	without	7731.2	11315.2	6348.8	9446.4
	D3	7577.6	10777.6	6118.4	9369.6
	rVV10	7654.4	11238.4	6579.2	9625.6

Table S14: Core-hours required for the calculation of phonon properties of MOF-5 and its Sr-substituted variant using different functionals and dispersion corrections.

Functional	Dispersion Correction	MOF-5	Sr-MOF-5
PBE	without	21708.8	–
	D3	49548.8	15897.6
	rVV10	22112.0	–
R2SCAN	without	43120.0	–
	D3	41184.0	6528.0
	rVV10	61334.4	–
PBE0	without	25369.6	–
	D3	26076.8	38201.6
	rVV10	20384.0	–
